# Management and outcomes of craniectomy and cranioplasty in pediatric intracranial infections: a multicenter cohort study

**DOI:** 10.1007/s00381-026-07149-4

**Published:** 2026-01-27

**Authors:** Artem Rafaelian, Sae-Yeon Won, Ulrich Sure, Phillipp Dammann, Laurèl Rauschenbach, Christian Senft, Nazife Dinc, Nazeer Aboud, Florian Ringel, Malte Ottenhausen, Daniel Scurtu, Wolfgang Deinsberger, Stefanie Kästner, Joe Jun Qiao Chen, Hartmut Vatter, Sevgi Sarikaya-Seiwert, Matthias Schneider, Joshua D. Bernstock, Silvia Hernández-Durán, Thomas M. Freiman, Florian Gessler, Daniel Dubinski

**Affiliations:** 1 Department of Neurosurgery, Rostock University Medical Center, Rostock, Germany; 2 Department of Neurosurgery and Spine Surgery, University Hospital Essen, University of Duisburg-Essen, Essen, Germany; 3 Department of Neurosurgery, Jena University Hospital, Friedrich Schiller University Jena, Jena, Germany; 4 Department of Neurosurgery, University Medical Center Mainz, Mainz, Germany; 5 Neurosurgical Department, Klinikum Kassel, Kassel, Germany; 6 Kassel School of Medicine Southampton, Southampton, Hampshire England; 7 Department of Neurosurgery, University Hospital Bonn, Bonn, Germany; 8 Department of Neurosurgery, Harvard Medical School, Brigham and Women’s Hospital, Boston, MA USA; 9 Department of Neurosurgery, LMU University Hospital of the Ludwig-Maximilians-University Munich, Munich, Germany; 10 Department of Neurosurgery, Heidelberg University Hospital, Heidelberg, Germany

**Keywords:** Craniectomy, Cranioplasty, Pediatric neurosurgery, Intracranial empyema

## Abstract

**Background:**

In recent years, there has been a noticeable increase in severe intracranial infections among pediatric patients, often requiring craniotomy or decompressive craniectomy. However, determining the optimal timing and material for cranioplasty in pediatric patients presents an ongoing challenge.

**Methods:**

We conducted a multicenter retrospective cohort study of pediatric patients treated between 2013 and 2024. A total of 76 children with intracranial infections were included; 37% of those underwent decompressive craniectomy. Of these, 21% subsequently received cranioplasty using various graft materials. Clinical, microbiological, radiological, and neurosurgical data were analyzed to assess risk factors, outcomes, and complications.

**Results:**

Craniectomy was significantly associated with the presence of sinusitis (*p* = 0.025) and paresis (*p = *0.006). *Streptococcus intermedius* was the predominant pathogen identified in the craniectomy group (*p* < 0.001). Cranioplasty was performed in 16 patients at a median of 6.9 months post-craniectomy, with a low complication rate (6.25%, one wound impairment). Functional outcome improved significantly in patients with hemicraniectomy after cranioplasty with favorable mRS scores at 3-month follow-up (*p* = 0.037).

**Conclusion:**

Craniectomy is frequently required in pediatric patients with severe intracranial infections and is followed by delayed cranioplasty with low complication rates in our cohort. Careful timing and individualized material selection are essential for safe and successful cranial reconstruction in this vulnerable population.

## Introduction

Intracranial infections such as sub- or epidural empyema, brain abscess, and ventriculitis in pediatric patients are life-threatening conditions that require urgent neurosurgical intervention [1]. Recent studies indicate a rising incidence of purulent intracranial complications in the pediatric population, including subdural and epidural empyemas [4, 23].

Management strategies for these severe infections vary widely, ranging from craniotomy with debridement to decompressive craniectomy combined with prolonged antibiotic therapy ^35^. When decompressive craniectomy is performed in pediatric neurosurgery, new problems arise with a skull defect that requires timely reconstruction. Following recovery and resolution of acute infection, the timing of cranioplasty, the choice of implant material, fixation techniques, and duration of antibiotic prophylaxis remain matters of debate [6].

In adult neurosurgery, cranioplasty is considered one of the most complication-prone procedures, with overall complication rates ranging from 15 to 41% [7–9]. In the pediatric setting, these rates are 10.5–42% and underscore the ongoing debate as to when and how cranioplasty should be performed [10, 11]. For adult patients undergoing surgical repair of cranial defects, autologous bone is considered the gold standard—provided it is available [12, 13].

Selecting the optimal implant material for cranioplasty in the pediatric population remains a significant challenge [14, 15]. The decision is further complicated in patients with a history of intracranial empyema, especially epidural empyema, where autologous bone may carry a significantly higher risk of reinfection.

Currently, when autologous bone is not available or not suitable for reconstruction, a variety of custom-made alloplastic materials are used. Among metallic options, titanium mesh is most commonly utilized due to its high mechanical strength and excellent resistance to infection [16]. In recent years, there has been growing interest in newer materials such as hydroxyapatite, polymethylmethacrylate (PMMA), polyetheretherketone (PEEK), and other acrylic-based composites. These materials are often easier to manufacture and, in some settings, are considered more cost-effective and simpler to handle in surgical practice [17, 18].

Determining the optimal timing for cranioplasty after central nervous system (CNS) infection is crucial, as children are particularly vulnerable both psychologically and socially, and prolonged cranial defects may impair development [19]. When considering timing, skull growth in relation to age must also be taken into account. In children, additional concerns include premature closure of cranial sutures, mismatch between skull growth and implant size, and the potential need for reoperation as the child grows [20–22].

Against this background, the present multicenter retrospective cohort study aimed to address two key objectives:

1. To identify clinical, radiological, and microbiological factors associated with the need for decompressive craniectomy in pediatric patients with intracranial infections;
2. To describe the timing, material selection, and outcomes of cranioplasty following craniectomy in this population.

## Methods

This multicenter study, conducted at six German university hospitals, was approved by the institutional review board of the University Medical Center Rostock (identification number 2024-0163). Informed consent was waived due to the retrospective design and use of anonymized data. Patients younger than 18 years diagnosed with intracranial infection (International Statistical Classification of Diseases-10-GM 2025 codes: G06.0–G06.2) between 2013 and 2024 were included. Inclusion criteria required that patients had undergone neurosurgical intervention for intracranial infection. Patients with a history of prior neurosurgical procedures before admission were excluded from the study to minimize the influence of potential postoperative infections. Additionally, patients with infections related to traumatic brain injury were also excluded due to their distinct pathophysiology. The study adhered to the Strengthening the Reporting of Observational Studies in Epidemiology (STROBE) guidelines.

**Fig. 1:**
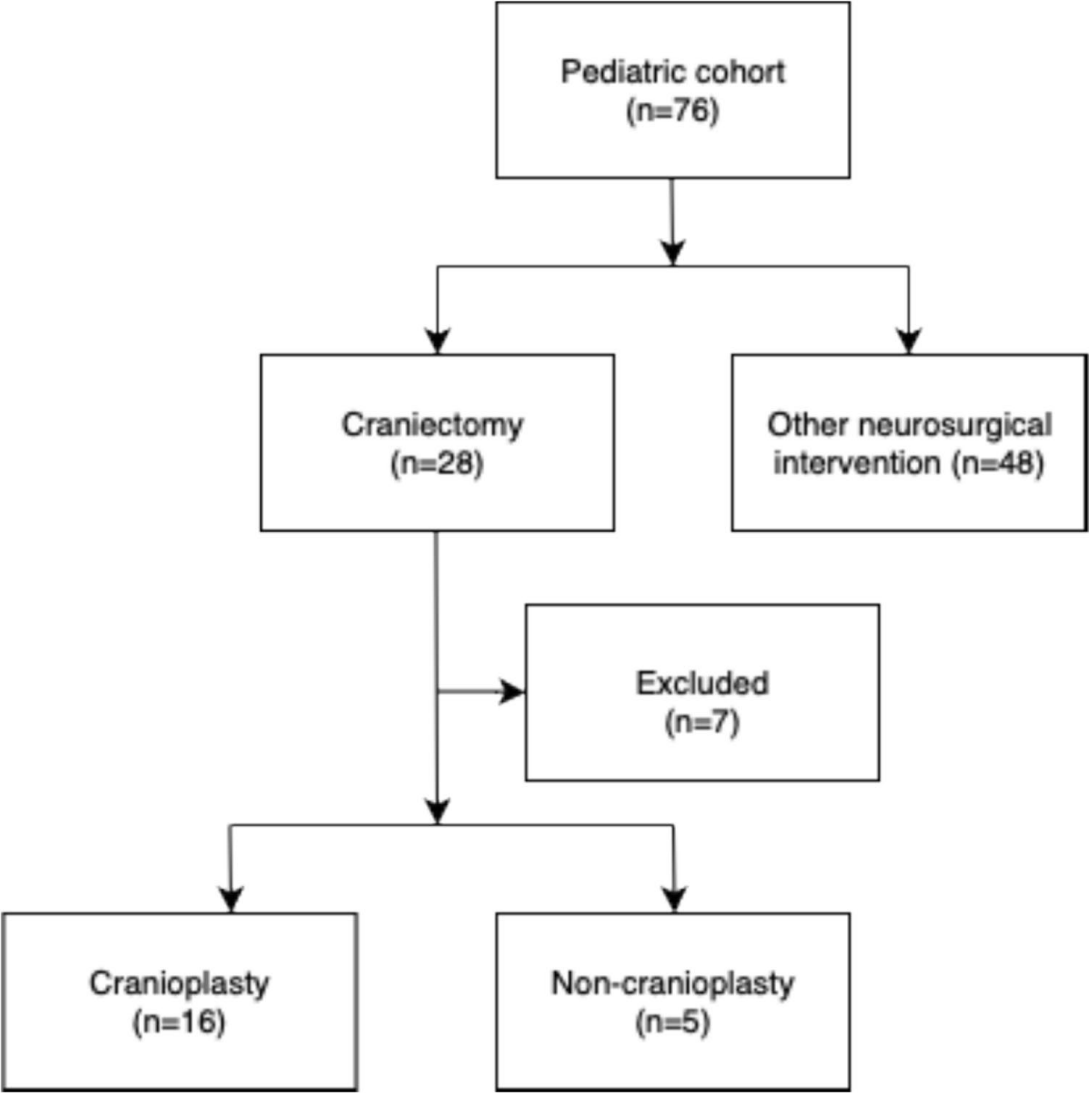
Flowchart of research group division. Patients excluded from further analysis after craniectomy included those with an mRS score of 6 (*n* = 2), those lost to follow-up (*n* = 2), and patients in whom cranioplasty was not performed for non-medical reasons, such as parental refusal or lack of consent (*n* = 3)

Collected data included patient age, sex, preoperative symptoms, preoperative antibiotic use, laboratory results, intraoperative intracranial swabs, prior surgeries, postoperative antibiotic management, type of neurosurgical procedure—including details on cranioplasty and its various types—postoperative complications, duration of hospitalization, pre- and postoperative MRI findings, and outcomes assessed by the pediatric modified Rankin Scale (mRS) at discharge and at 3 months follow-up. The pediatric mRS evaluates functional outcomes in children following neurological injury, ranging from 0 (no symptoms) to 6 (death), and is adapted to consider developmental milestones. Scores of 0 to 2 were defined as a “good outcome,” indicating preserved independence and age-appropriate functioning. Postoperative complications included the development of hematoma, edema with subsequent midline shift, and wound healing disorders. Patient group stratification is illustrated in Fig. 1.

### Statistical analysis

Univariate and multivariate analyses were performed using logistic regression (SPSS Statistics version 29, IBM Corp., Armonk, NY, USA). Odds ratios (ORs) with 95% confidence intervals (CIs) were calculated. For categorical variables, Fisher’s exact test was applied when appropriate. Spearman’s rank correlation was used to assess associations between continuous variables. Results with a *p*-value of ≤ 0.05 were considered statistically significant. Variables significant in univariate analysis (*p* ≤ 0.05) were included in multivariable logistic regression. All additional statistical analyses were performed using GraphPad Prism version 10 (GraphPad Software, California, USA).

## Results

A total of 76 patients were included in this multicenter study. 1. The patient cohort was divided into two groups: 28 patients (36.8%) who underwent craniectomy and 48 patients (63.2%) who received other, less “invasive” neurosurgical interventions. The median age was 8 years (IQR 2–11), and the median length of hospital stay was 31 days (IQR 18–38). Headache and fever were the most common presenting symptoms (55.2% and 63.1%). At admission, median CRP was markedly elevated (100 mg/L; IQR 24.8–206). Postoperative complications occurred in 11 patients (14.4%), including postoperative hematoma, postoperative swelling with midline shift, and wound healing disorders. Ventriculoperitoneal shunt was required in six patients (7.9%) during the hospital stay. A favorable outcome (pediatric mRS score of 0–2) was achieved in 55 patients (72.3%) at discharge and in 63 patients (82.9%) at the 3-month follow-up. A complete overview of demographic and clinical characteristics is presented in Table 1.

**Table 1:** Baseline demographic, clinical, and laboratory characteristics of the pediatric intracranial infection cohort

Variable	Cohort: 76
Age, median (IQR)	8 (2–11)
Male, *n* (%)	55 (72.4%)
Duration of hospitalization in days (IQR)	31 (18–38)
Preoperative state	
Headache, *n* (%)	42 (55.2%)
Fever, *n* (%)	48 (63.1%)
Neck stiffness, *n* (%)	21 (27.6%)
Sinusitis, *n* (%)	26 (34.2%)
Otitis, *n* (%)	21 (27.6%)
Preoperative antibiotics, *n* (%)	50 (65.8%)
Neurological deficit	
GCS, median, (IQR)	14 (11–15)
Aphasia, *n* (%)	9 (11.8%)
Paresis, *n* (%)	21 (27.6%)
Seizure, *n *(%)	20 (26.3%)
Systemic signs of infection	
Tmax, °C median (IQR)	38.6 (37.2–39.5)
CRP, mg/L, median (IQR)	100 (24.8–206)
PCT, ng/mL, median (IQR)	0.3 (0.09–2.32)
Preoperative MRI	
Contrast enhancing,* n *(%)	63 (82.9%)
DWI/ADC positive, *n* (%)	63 (82.9%)
Epidural abscess, *n* (%)	19 (25%)
Subdural empyema, *n* (%)	22 (28.9%)
Cerebral abscess, *n* (%)	17 (22.4%)
Meningitis, *n* (%)	16 (21.1%)
Ventricular extension, *n* (%)	7 (9.2%)
Midline shift, *n* (%)	18 (23.7%)
Neurosurgical intervention	
Burr hole, *n* (%)	30 (39.4%)
EVD, *n* (%)	4 (5.2%)
Craniotomy, *n* (%)	14 (18.4%)
Craniectomy, *n* (%)	28 (36.8%)
Perioperatives management	
Postoperative complication, *n* (%)	11 (14.4%)
Intraoperative intracranial swabs, *n* (%)	60 (78.9%)
VP shunt implantation, *n* (%)	6 (7.9%)
Prior ENT surgery, *n* (%)	37 (48.7%)
Intraoperative intracranial germs	
*Streptococcus intermedius*, *n* (%)	21 (27.6%)
*Staphylococcus aureus*, *n* (%)	7 (9.2%)
*Streptococcus pneumoniae*, *n* (%)	8 (10.5%)
*Haemophilus influenzae*, n (%)	4 (5.2%)
Others, *n* (%)	20 (26.3%)
Pediatric mRS at discharge	
0–2	55 (72.3%)
3–5	15 (19.7%)
6	6 (7.9%)
Pediatric mRS at 3 months follow-up	
0–2	63 (82.9%)
3–5	6 (7.9%)
6	7 (9.2%)

*GCS*, Glasgow Coma Scale; *Tmax*, maximum temperature (°C); *CRP*, C-reactive protein (mg/L); *PCT*, procalcitonin (ng/mL); *DWI*/*ADC*, diffusion-weighted imaging/apparent diffusion coefficient; *EVD*, external ventricular drain; *VP*, ventriculoperitoneal; *ENT*, ear, nose, and throat surgery; *mRS*, pediatric modified Rankin Scale; data are presented as median (IQR) for continuous variables and number (%) for categorical variables

The median age of patients in the craniectomy group was significantly higher (9 years, IQR 3–12.25) compared to the non-craniectomy group (6.5 years, IQR 1–10.25; *p* = 0.034). The craniectomy group had a significantly higher proportion of male patients (89.3% vs. 62.5%; OR 5.0, 95% CI 1.32–18.95, *p* = 0.009), although this difference did not remain significant in multivariate analysis (*p* = 0.163). Although the median hospital stay was slightly longer in the craniectomy group (32 vs. 30.5 days), this difference was not statistically significant (*p* = 0.177) (Table 2).

**Table 2:** Results of univariate and multivariate analyses comparing clinical and treatment variables between patients with and without craniectomy

	Craniectomy	Other neurosurgical intervention	Univariate		Multivariate	
Cohort	28	48	OR 95% CI	***p***-value	OR 95% CI	***p***-value
Age, median (IQR)	9 (3–12.25)	6.5 (1–10.25)		**0.034**	1.074 (0.87–1.32)	0.5
Male, *n* (%)	25 (89.3%)	30 (62.5%)	5 (1.32–18.95)	**0.009**	0.287 (0.05–1.66)	0.163
Duration of hospitalization in days (IQR)	32 (20–45,25)	30.5 (14–37)		0.177		
Preoperative state						
Headache, *n* (%)	17 (60.7%)	25 (52.1%)	1.42 (0.55–3.66)	0.313		
Fever, *n* (%)	15 (53.5%)	33 (68.7%)	0.53 (0.21–1.37)	0.141		
Neck stiffness, *n* (%)	8 (28.5%)	13 (27.1%)	1.08 (0.38–3.04)	0.545		
Sinusitis, *n* (%)	14 (50%)	12 (25%)	3 (1.12–8.06)	**0.025**	1.095 (0.89–1.34)	0.302
Otitis, *n* (%)	5 (17.9%)	16 (33.3%)	0.44 (0.11–1.36)	0.116		
Preoperative antibiotics, *n* (%)	19 (67.8%)	31 (64.5%)	1.16 (0.43–3.11)	0.487		
Neurological deficit at admission						
GCS, median, (IQR)	13.5 (11–14)	14 (12–15)		0.449		
Aphasia, *n* (%)	5 (17.8%)	4 (8.3%)	2.39 (0.58–9.78)	0.19		
Paresis, *n* (%)	13 (46.4%)	8 (16.6%)	4.33 (1.5–12.53)	**0.006**	0.475 (0.11–2.12)	0.329
Seizure, *n* (%)	5 (17.9%)	15 (31.3%)	0.48 (0.15–1.51)	0.156		
Systemic signs of infection						
Tmax, °C median (IQR)	37.6 (37.18–39.13)	38.5 (37.2–39.65)		0.174		
CRP, mg/L, median (IQR)	94 (20–206)	80.3 (26.5- 194)		0.495		
PCT, ng/mL, median (IQR)	0.492 (0.09–9.27)	0.21 (0.1–3.11)		0.138		
Preoperative MRI						
Contrast enhancing, *n* (%)	23 (82.1%)	40 (83.3%)	0.92 (0.27–3.15)	0.564		
DWI/ADC positive, *n* (%)	24 (85.7%)	39 (81.2%)	1.38 (0.38–4.99)	0.435		
Epidural abscess, *n* (%)	5 (17.8%)	14 (29.2%)	0.53 (0.17–1.67)	0.206		
Subdural empyema, *n* (%)	11 (39.3%)	11 (22.9%)	2.18 (0.79–5.99)	0.105		
Cerebral abscess, *n* (%)	7 (25%)	10 (20.8%)	1.27 (0.42–3.82)	0.441		
Meningitis, *n* (%)	5 (17.8%)	11 (22.9%)	0.73 (0.23–2.38)	0.414		
Ventricular involement, *n* (%)	0 (0%)	7 (14.6%)	-	**0.033**		0.99
Midline shift, *n* (%)	9 (32.1%)	9 (18.8%)	2.05 (0.71–6.01)	0.148		
Perioperatives management						
Postoperative complication, *n* (%)	8 (28.6%)	3 (6.3%)	6 (1.44–25.01)	**0.011**	0.276 (0.04–1.79)	0.177
Intraoperative intracranial swabs, *n* (%)	26 (92.8%)	34 (70.8%)	5.35 (1.17–25.66)	**0.02**		
Total number of operations, median (IQR)	2 (1–3)	1 (1–1)		0.156		
VP shunt implantation, *n* (%)	3 (10.7%)	3 (6.25%)	1.8 (0.34–9.59)	0.388		
Prior ENT surgery, *n* (%)	18 (64.2%)	19 (39.5%)	2.74 (1.05–7.22)	**0.032**	0.741 (0.12–4.76)	0.752
Duration of antibiotic treatment, median (IQR)	6 (5.5–8)	6 (4–7.5)		0.301		
Intraoperative intracranial germs						
*Streptococcus intermedius*, *n* (%)	15 (53.5%)	6 (12.5%)	8.08 (2.61–25.07)	**0.0001**	6.591 (1.6–27.16)	**0.009**
*Staphylococcus aureus*, *n* (%)	3 (10.7%)	4 (8.3%)	1.32 (0.27–6.38)	0.513		
*Streptococcus pneumoniae*, *n* (%)	2 (7.1%)	6 (12.5%)	0.54 (0.11 −2.87)	0.375		
*Haemophilus influenzae*, *n *(%)	1 (3.5%)	3 (6.3%)	0.56 (0.06–5.62)	0.529		
Others, *n* (%)	5 (17.9%)	15 (31.3%)	0.48 (0.15–1.51)	0.157		
Pediatric mRS at discharge						
0–2	20 (71.4%)	35 (72.9%)	0.93 (0.33–2.62)	0.545		
3–5	6 (21.4%)	9 (18.8%)	1.18 (0.37–3.76)	0.499		
6	2 (7.1%)	4 (8.3%)	0.85 (0.15–4.95)	0.611		
Pediatric mRS at 3 months follow-up						
0–2	24 (85.7%)	39 (81.2%)	1.384 (0.38–4.99)	0.435		
3–5	2 (7.1%)	4 (8.3%)	0.8462 (0.15–4.94)	0.611		
6	2 (7.1%)	5 (10.4%)	0.662 (0.12–3.66)	0.708		

*CRP*, C-reactive protein (mg/L); DWI/ *ADC*, diffusion-weighted imaging/apparent diffusion coefficient; *ENT*, ear, nose, and throat surgery; *EVD*, external ventricular drain; *GCS*, Glasgow Coma Scale; *mRS*, pediatric modified Rankin Scale; *PCT*, procalcitonin (ng/mL); Tmax, maximum temperature (°C); *VP*, ventriculoperitoneal; *OR*, odds ratio; 95% *CI*, 95% confidence interval. Data are presented as median (IQR) for continuous variables and number (%) for categorical variables

### Preoperative clinical and laboratory features

Headache and fever were the most common preoperative symptoms in both groups, with headache reported in 60.7% of the craniectomy group vs. 52.1% (*p* = 0.313) and fever in 53.5% vs. 68.7% (*p* = 0.141), respectively. Sinusitis was significantly more prevalent in patients undergoing craniectomy (50% vs. 25%, OR 3.0, 95% CI 1.12–8.06, *p* = 0.025). There were no significant differences in the rates of neck stiffness, otitis, or preoperative antibiotic use between the groups. Neurological deficits such as paresis were significantly more frequent in the craniectomy group (46.4% vs. 16.6%, OR 4.33, 95% CI 1.5–12.53, *p* = 0.006), while aphasia and seizures did not differ significantly. GCS at admission scores were slightly lower in the craniectomy group (median 13.5 vs. 14), though this difference was not significant. Systemic signs of infection such as maximum temperature (Tmax), CRP, and procalcitonin (PCT) levels showed no significant difference between groups, although CRP levels were higher in the craniectomy group (median 94 vs. 80.3 mg/L).

### Radiological findings

MRI findings revealed higher rates of subdural empyema (39.3% vs. 22.9%) and midline shift (32.1% vs. 18.8%) in the craniectomy group, although these were not statistically significant. Ventricular involvement was found only in the non-craniectomy group (14.6%, *p* = 0.033), suggesting that different infection localizations may influence surgical decisions.

### Perioperative and microbiological data

Postoperative complications occurred significantly more often in the craniectomy group (28.6% vs. 6.3%; OR 6.0, 95% CI 1.44–25.01, *p* = 0.011), though this was not significant in multivariate analysis (hematoma, edema with subsequent midline shift, and wound impairment). The number of positive intraoperative intracranial swabs was also higher (92.8% vs. 70.8%, *p* = 0.02). Patients with craniectomy more often had a history of prior ENT surgery (64.2% vs. 39.5%, *p* = 0.032). The predominant pathogen isolated in the craniectomy group was *Streptococcus intermedius* (53.5% vs. 12.5%; OR 8.08, *p* < 0.0001), which remained significant in multivariate analysis (OR 6.591, 95% CI 1.6–27.16, *p* = 0.009). Other bacteria such as *Staphylococcus aureus* and *Haemophilus influenzae* were observed in low frequencies without significant differences between groups.

### Outcomes

Despite a higher complication rate, early and late outcomes were similar across both groups. At discharge, a good outcome (mRS 0–2) was achieved in 71.4% of craniectomy patients vs. 72.9% without craniectomy (*p* = 0.545), and at 3 months follow-up in 85.7% vs. 81.2% (*p* = 0.435), respectively. There was no significant difference in mortality between *two cohorts* (7.1% vs. 10.4%, respectively, *p* = 0.611).

### Timing and material selection in pediatric cranioplasty

Among 16 pediatric patients who underwent cranioplasty following intracranial infection and craniectomy, the median age was 10 years (IQR 5.5–14), with 75% being male. The median cranial defect size was 57.2 cm^2^ (IQR 15.9–82.9), and the median time from craniectomy to cranioplasty was 6.9 months (IQR 3.93–17.3). Hospital stay following cranioplasty was relatively short, with a median duration of 4 days (IQR 3–5) (Table 3).

**Table 3:** Patients who received cranioplasty

	Cohort: 16
Age, median (IQR)	10 (5.5–14)
Male n (%)	12 (75%)
Defect size (cm^2^) (IQR)	57.2 (15.9–82.9)
time to cranioplasty, median (IQR) (months)	6.9 (3.93–17.3)
Duration of hospitalization in days (IQR)	4 (3–5)
Graft type used	
Autograft	3 (18.8%)
PEEK	4 (25%)
Titanium	4 (25%)
Polyethylene	2 (12.5%)
Polymethylmethacrylat	2 (12.5%)
Glass fiber reinforced polymer	1 (6.25%)
Method of cranioplasty fixation	
Titanium plates	10 (62.5%)
Mini screws	5 (31.25%)
Bone sutures	1 (6.25%)
Postoperative monitoring	
Postoperative CT scan	14 (87.5%)
Postoperative MRI	2 (12.5%)
Postoperative complication	
Wound healing disorder	1 (6.25%)
Pediatric mRS at discharge	
0–1	11 (68.8%)
2	3 (18.8%)
3	1 (6.3%)
4	1 (6.3%)
Pediatric mRS at 3 months follow-up	
0–1	12 (75%)
2	3 (18.8%)
3	1 (6.3%)
Observation time, median (IQR) (month)	12.7 (4.5–29.6)

*CT*, computed tomography; *MRI*, magnetic resonance imaging; *mRS*, modified Rankin Scale; *PEEK*, polyether ether ketone; *GFRC*, glass fiber reinforced polymer; cm2, square centimeters. Data are presented as median (IQR) for continuous variables and number (%) for categorical variables

A variety of reconstruction materials were used for cranioplasty. Synthetic grafts were most frequently applied, with PEEK and titanium each used in 25% of cases, while autologous bone was utilized in 18.8% of patients. Other materials included polyethylene (12.5%), polymethylmethacrylat (12.5%), and glass fiber reinforced polymer (6.25%). Fixation techniques predominantly involved titanium plates (62.5%), followed by mini screws (31.25%) and bone sutures (6.25%). Postoperative monitoring was performed mainly via CT imaging (87.5%), while MRI was used in only 12.5% of cases. Postoperative complications were rare, with only one case (6.25%) of wound impairment reported. At discharge, 68.8% of patients had a favorable outcome defined as a pediatric mRS score of 0–1. This proportion increased to 75% at 3 months follow-up, demonstrating significant improvement in neurological status (*p* = 0.037). Median observation time after cranioplasty was 12.7 months (IQR 4.5–29.6).

### Patients who did not undergo cranioplasty

Among the 12 pediatric patients who did not receive cranioplasty after craniectomy, the median age was 6.5 years (IQR 2.5–10.25), and the majority were male (91.6%). The median cranial defect size in this group was significantly smaller than in those who received cranioplasty, measuring 9.4 cm^2^ (IQR 5.6–13.2). In nearly half of the cases (41.6%), the cranial defect was located in the posterior fossa. In 2 cases (16.7%), the patients died during hospitalization. Three families (25%) withdrew consent for the procedure, and in another 2 cases (16.7%), follow-up was not possible due to loss of contact (Table 4).

**Table 4:** Patients who did not undergo cranioplasty

	Cohort: 12
Age, median (IQR)	6.5 (2.5–10.25)
Male, *n* (%)	11 (91.6%)
Defect size (cm^2^) (IQR)	9.4 (5.6–13.2)
Reasons why cranioplasty was not performed	
Defect of the posterior cranial fossa	5 (41.6%)
Fatal outcome	2 (16.7%)
Patients consent withdrawn	3 (25%)
Lost to follow-up	2 (16.7%)

Data are presented as median (IQR) for continuous variables and number (%) for categorical variables

## Discussion

This multicenter study provides one of the first systematic assessments of decompressive craniectomy and subsequent cranioplasty in pediatric patients with intracranial infections. Among 76 patients, 28 patients (36.8%) underwent decompressive craniectomy as part of their treatment, and of these, 16 (57.1%) subsequently received cranioplasty. Our findings indicate that children who required craniectomy were significantly older, presented with severe neurological deficits, and frequently had underlying sinusitis. These factors likely reflect a more aggressive infectious process with increased intracranial pressure rather than independent triggers for surgical decision-making. Importantly, these variables should be interpreted as markers of underlying disease severity rather than direct predictors guiding the choice of craniectomy.

Although decompressive craniectomy is a well-established method in adult neurosurgery, especially in cases of traumatic brain injury with malignant brain edema, its role in intracranial infections remains insufficiently studied, especially in pediatric patients. Most existing literature sources focus on the adult population, with craniectomy being performed in approximately 33.4% of cases of severe CNS infections [23, 24]. Consequently, the decision to perform craniectomy is typically made on an individual case basis and relies on multidisciplinary discussion between neurosurgeons, pediatric intensivists, and pediatricians.

Interestingly, intraoperative identification of *Streptococcus intermedius* was significantly more common in children in the craniectomy cohort, supporting previous observations of its association with aggressive infection patterns and multiloculated abscess formation [25]. *Streptococcus intermedius* is a β-hemolytic Gram-positive member of the *Streptococcus anginosus* group. The process of *S. intermedius* brain abscess formation begins with tissue damage mediated by its binding to human fibronectin and laminin. This interaction triggers the release of interleukin-8 (IL-8) from monocytes, leading to neutrophil chemotaxis and the accumulation of proinflammatory cytokines that cause further tissue damage. Moreover, *S. intermedius* expresses hydrolytic enzymes responsible for tissue liquefaction and pus formation [26]. Its polysaccharide capsule helps the bacterium to avoid phagocytosis, encapsulate within pus clusters, and form abscesses [27]. Taking the latter into consideration, the initial empiric antibiotic therapy should primarily target Gram-positive bacteria.

### Craniectomy and postoperative complications

Early studies in adults have shown that the prevalence of complications in adults undergoing craniectomy can reach 13.4% [28]. Our findings align with this, showing a higher incidence of postoperative complications in the craniectomy group compared to those undergoing less invasive procedures. Nonetheless, functional outcomes at discharge and 3-month follow-up were comparable between groups, suggesting that even patients with severe disease can achieve good recovery with appropriate management.

### Selection of timing and material for cranioplasty

Regarding cranioplasty, earlier studies in children report complication rates ranged 10.5–42%, especially when autologous bone is used after infection [10, 11]. Our low complication rate (6.25%) may reflect careful selection of timing and cases or the small study group. The median follow-up after cranioplasty was just over 1 year, which is sufficient to recognize both early and late complications.

The literature also emphasizes the fact that there is still no consensus on the ideal timing for cranioplasty, especially after primary CNS infection [29, 30]. Among patients who underwent cranioplasty, no single graft type predominated. A variety of materials—including autografts, PEEK, titanium, polyethylene, Palacos, and glass fiber-reinforced polymers—were used, highlighting the lack of consensus on the optimal implant material in children after CNS infection. Median time to cranioplasty was 6.9 months (IQR 3.9–17.3), and only one complication (6.25%) occurred, suggesting that a delayed and individualized approach to reconstruction may be safe and effective. This relatively delayed timing may reflect several factors: the need for prolonged courses of antibiotic therapy after hospital discharge, extended periods of neurological and physical rehabilitation, and the lack of standardized clinical guidelines for pediatric post-infectious cranioplasty. Therefore, decisions are often made on an individual case basis, and treating teams often take a certain “defensive” approach with children to ensure with a high degree of certainty that the infection has been completely cured before proceeding with reconstruction. It is possible that even in cases of isolated subdural empyema secondary to ENT infections, there is partial involvement of the cranial bone. In such situations, both treatment and subsequent cranioplasty may reasonably be managed according to the principles of osteomyelitis. Previous studies have shown that reconstructive cranial surgery should ideally be delayed for at least 6 months after the initial infection to minimize the risk of reinfection and ensure complete eradication of the pathogen [31]. In contrast, Lajthia et al. reported favorable outcomes with immediate replacement of the autologous bone graft after empyema drainage in children, suggesting that early cranioplasty may be feasible in carefully selected cases [32]. Nevertheless, our findings, consistent with prior reports recommending delayed reconstruction, are in line with a cautious and individualized approach, although definitive conclusions cannot be drawn from this cohort.

Despite the complexity of treating pediatric skull defects following infection, outcomes in our cohort were generally favorable. Most children underwent postoperative CT imaging to rule out complications, although no postoperative hemorrhages or implant-related complications were observed. Given the radiation risks in children and the low complication rate, routine CT follow-up might be reconsidered in favor of MRI, ultrasound (for transparent implants), or even plain radiographs where appropriate.

In addition to neurological recovery, delayed cranial reconstruction may have psychosocial implications, particularly in school-aged children [10, 19]. In our cohort, improvement in pediatric mRS scores at 3 months post-cranioplasty likely reflects overall neurological recovery; however, potential psychosocial benefits cannot be directly assessed in this retrospective analysis and should be interpreted cautiously.

## Limitations

This study has a number of limitations that should be considered when interpreting the results. First, its retrospective design introduces selection bias. Second, although this represents one of the larger multicenter pediatric cohorts on this topic, the overall sample size—and particularly the size of relevant subgroups—remains limited, reducing statistical power and the robustness of multivariable analyses. Third, heterogeneity in infections, surgical strategies, and implant materials reduces cohort homogeneity. In addition, the lack of a matched comparison group precludes definitive conclusions regarding the independent effect of cranioplasty on neurological recovery, as improvements may also reflect ongoing post-infectious and post-surgical neurological recovery. Future prospective studies with larger patient groups, standardized treatment protocols, and longer follow-up of neuropsychological development are needed to confirm and expand our findings.

## Conclusion

Our study highlights the complexity of surgical decisions in pediatric intracranial infections. The need for craniectomy is often driven by preoperative deficits, overall clinical condition, and intraoperative findings that cannot always be predicted in advance, such as extensive subdural empyema and increased intracranial pressure. Despite the severity of these cases, most patients achieved favorable recovery within 3 months, indicating that decompressive surgery can lead to good outcomes. Cranioplasty after infection should follow an individualized approach: various materials proved feasible in our cohort, and timing should carefully balance infection control with neurodevelopmental and psychosocial considerations.

## Data Availability

The datasets generated during and/or analyzed during the current study are available from the corresponding author on reasonable request.

## References

[1] Legrand M, Roujeau T, Meyer P, Carli P, Orliaguet G, Blanot S (2009) Paediatric intracranial empyema: differences according to age. Eur J Pediatr 168(10):1235–1241. 10.1007/s00431-008-0918-4 19137324 10.1007/s00431-008-0918-4

[2] Ooi SZY, Sichimba D, Dalle DU et al (2021) Management and outcomes of paediatric intracranial suppurations in low- and middle-income countries: a scoping review. Front Surg. 10.3389/fsurg.2021.690895 34466410 10.3389/fsurg.2021.690895 PMC8403063

[3] Dubinski D, Won SY, Rafaelian A, et al. Pediatric intracranial infections requiring neurosurgery: a German multicenter analysis of 10-year trends pre–COVID-19 and post–COVID-19. *Neurosurgery*. Published online July 18, 2025. 10.1227/neu.0000000000003640 10.1227/neu.0000000000003640 PMC12875630 40693723

[4] Liu Y, Bai Z, Yang T et al (2024) Changes in the epidemiology of pediatric brain abscesses pre- and post-COVID-19 pandemic: a single-center study. BMC Pediatr. 10.1186/s12887-024-05082-6 39306664 10.1186/s12887-024-05082-6 PMC11416000

[5] Boukassa L, Ngackosso OB, Kinata SB, Mbaki HBE (2020) Intracranial suppurations in Africa: systematic review. Open J Mod Neurosurg 10(02):244–253. 10.4236/ojmn.2020.102026

[6] Klieverik VM, Miller KJ, Han K Sen, et al. Cranioplasties following craniectomies in children—a multicenter, retrospective cohort study. *Child’s Nervous System*. 2019;35(9):1473–1480. 10.1007/s00381-018-4024-2 10.1007/s00381-018-4024-2 30554262

[7] Sauvigny T, Giese H, Höhne J et al (2022) A multicenter cohort study of early complications after cranioplasty: results of the German Cranial Reconstruction Registry. J Neurosurg 137(2):591–598. 10.3171/2021.9.JNS211549 34920418 10.3171/2021.9.JNS211549

[8] Armstrong RE, Ellis MF (2019) Determinants of 30-day morbidity in adult cranioplasty: an ACS-NSQIP analysis of 697 cases. Plast Reconstr Surg Glob Open 7(12):E2562. 10.1097/GOX.0000000000002562 32537306 10.1097/GOX.0000000000002562 PMC7288897

[9] Mee H, Anwar F, Timofeev I et al (2022) Cranioplasty: a multidisciplinary approach. Front Surg. 10.3389/fsurg.2022.864385 35656088 10.3389/fsurg.2022.864385 PMC9152220

[10] Rogers SJ, Browne AL, Vidovich M, Honeybul S (2011) Defining meaningful outcomes after decompressive craniectomy for traumatic brain injury: existing challenges and future targets. Brain Inj 25(7–8):651–663. 10.3109/02699052.2011.580316 21561294 10.3109/02699052.2011.580316

[11] Piedra MP, Thompson EM, Selden NR, Ragel BT, Guillaume DJ (2012) Optimal timing of autologous cranioplasty after decompressive craniectomy in children: clinical article. J Neurosurg Pediatr 10(4):268–272. 10.3171/2012.6.PEDS1268 22861195 10.3171/2012.6.PEDS1268

[12] Cabbad NC, Stalder MW, Arroyave A, Wolfe EM, Wolfe SA (2019) Autogenous bone cranioplasty: review of a 42-year experience by a single surgeon. Plast Reconstr SurgLippincott Williams and Wilkins 143(6):1713–1723. 10.1097/PRS.0000000000005677 10.1097/PRS.0000000000005677 31136489

[13] Sundseth J, Sundseth A, Berg-Johnsen J, Sorteberg W, Lindegaard KF (2014) Cranioplasty with autologous cryopreserved bone after decompressive craniectomy. Complications and risk factors for developing surgical site infection. Acta Neurochir (Wien) 156(4):805–811. 10.1007/s00701-013-1992-6 24493001 10.1007/s00701-013-1992-6 PMC3956933

[14] Still MEH, Samant S, Alvarado A, Neal D, Governale LS, Ching JA (2023) Considerations for choice of cranioplasty material for pediatric patients. Pediatr Neurosurg 58(1):1–7. 10.1159/000528543 36477047 10.1159/000528543 PMC10064395

[15] Aprianto DR, Parenrengi MA, Utomo B, Fauzi AA, Subagio EA, Suryawan A (2022) Comparison of autograft and implant cranioplasty in pediatrics: a meta-analysis. Surg Neurol Int. 10.25259/SNI_1204_2021 36324924 10.25259/SNI_1204_2021 PMC9610369

[16] Ma IT, Symon MR, Bristol RE et al (2018) Outcomes of titanium mesh cranioplasty in pediatric patients. J Craniofac Surg 29(1):99–104. 10.1097/SCS.0000000000004045 29049146 10.1097/SCS.0000000000004045

[17] Zaccaria L, Tharakan SJ, Altermatt S (2017) Hydroxyapatite ceramic implants for cranioplasty in children: a single-center experience. Childs Nerv Syst 33(2):343–348. 10.1007/s00381-016-3327-4 28013335 10.1007/s00381-016-3327-4

[18] O’Reilly EB, Barnett S, Madden C, Welch B, Mickey B, Rozen S (2015) Computed-tomography modeled polyether ether ketone (PEEK) implants in revision cranioplasty. J Plast Reconstr Aesthet Surg 68(3):329–338. 10.1016/j.bjps.2014.11.001 25541423 10.1016/j.bjps.2014.11.001

[19] Ballestero MFM, Furlanetti LL, Augusto LP, Chaves PHC, Santos MV, de Oliveira RS (2019) Decompressive craniectomy for severe traumatic brain injury in children: analysis of long-term neuropsychological impairment and review of the literature. Childs Nerv Syst 35(9):1507–1515. 10.1007/s00381-019-04274-1 31264065 10.1007/s00381-019-04274-1

[20] Governale LS (2015) Craniosynostosis. Pediatr Neurol 53(5):394–401. 10.1016/j.pediatrneurol.2015.07.006 26371995 10.1016/j.pediatrneurol.2015.07.006

[21] Frassanito P, Beez T (2024) Cranial Repair in Children: Techniques, Materials, and Peculiar Issues. <book-title update="added">Advances and technical standards in neurosurgery: volume 49. pp 307–326. 10.1007/978-3-031-42398-7_14 10.1007/978-3-031-42398-7_14 38700690

[22] Martin KD, Franz B, Kirsch M et al (2014) Autologous bone flap cranioplasty following decompressive craniectomy is combined with a high complication rate in pediatric traumatic brain injury patients. Acta Neurochir (Wien) 156(4):813–824. 10.1007/s00701-014-2021-0 24532225 10.1007/s00701-014-2021-0

[23] Nathoo N, Syed FCS, Nadvi S, Gouws E, Van Dellen JR. *Craniotomy improves outcomes for cranial subdural empyemas: computed tomography-era experience with 699 patients*. 10.1097/00006123-200110000-00017 11564248

[24] Mat Nayan SA, Mohd Haspani MS, Abd Latiff AZ, Abdullah JM, Abdullah S (2009) Two surgical methods used in 90 patients with intracranial subdural empyema. J Clin Neurosci 16(12):1567–1571. 10.1016/j.jocn.2009.01.036 19793660 10.1016/j.jocn.2009.01.036

[25] Brouwer MC, Coutinho JM, Van De Beek D (2014) Clinical characteristics and outcome of brain abscess :systematic review and meta-analysis. Neurology 82(9):806–813. 10.1212/WNL.0000000000000172 24477107 10.1212/WNL.0000000000000172

[26] Issa E, Salloum T, Tokajian S (2020) From normal flora to brain abscesses: a review of Streptococcus intermedius. Front Microbiol. 10.3389/fmicb.2020.00826 32457718 10.3389/fmicb.2020.00826 PMC7221147

[27] Kanamori S, Kusano N, Shinzato T, Saito A (2004) The role of the capsule of the Streptococcus milleri group in its pathogenicity. J Infect Chemother 10(2):105–109. 10.1007/s10156-004-0305-7 15160304 10.1007/s10156-004-0305-7

[28] Kurland DB, Khaladj-Ghom A, Stokum JA et al (2015) Complications associated with decompressive craniectomy: a systematic review. Neurocrit Care 23(2):292–304. 10.1007/s12028-015-0144-7 26032808 10.1007/s12028-015-0144-7 PMC4704457

[29] Malcolm JG, Rindler RS, Chu JK, Grossberg JA, Pradilla G, Ahmad FU (2016) Complications following cranioplasty and relationship to timing: a systematic review and meta-analysis. J Clin Neurosci 33:39–51. 10.1016/j.jocn.2016.04.017 27499122 10.1016/j.jocn.2016.04.017

[30] Zheng F, Xu H, von Spreckelsen N et al (2018) Early or late cranioplasty following decompressive craniotomy for traumatic brain injury: a systematic review and meta-analysis. J Int Med Res 46(7):2503–2512. 10.1177/0300060518755148 29779445 10.1177/0300060518755148 PMC6124291

[31] Kwiecien GJ, Aliotta R, Bassiri Gharb B, Gastman B, Zins JE (2019) The timing of alloplastic cranioplasty in the setting of previous osteomyelitis. Plast Reconstr Surg 143(3):853–861. 10.1097/PRS.0000000000005363 30601322 10.1097/PRS.0000000000005363

[32] Lajthia O, Chao JW, Mandelbaum M et al (2018) Efficacy of immediate replacement of cranial bone graft following drainage of intracranial empyema. J Neurosurg Pediatr 22(3):317–322. 10.3171/2018.3.PEDS17509 29932367 10.3171/2018.3.PEDS17509

